# Probing internal continua and atomic ultrafast charge transfer within size-controlled nanoparticles by post-collision interaction in core-hole clock spectroscopy

**DOI:** 10.3762/bjnano.17.33

**Published:** 2026-04-07

**Authors:** Johannes Lütgert, Erika Giangrisostomi, Nomi L A N Sorgenfrei, Alexander Föhlisch

**Affiliations:** 1 Institute Methods and Instrumentation for Synchrotron Radiation Research, Helmholtz-Zentrum Berlin, Albert-Einstein-Straße 15, 12489 Berlin, Germany; 2 Institute of Physics and Astronomy, University of Potsdam, Karl-Liebknecht-Straße 24/25, 14476 Potsdam-Golm, Germany

**Keywords:** core-hole clock, nanoparticles, post-collision-interaction, quantum dots, resonant Auger spectroscopy

## Abstract

This study investigates size-controlled, quantum-confined CdSe/ZnS core–shell quantum dots using core-hole clock spectroscopy in combination with post-collision interaction (PCI) line shape analysis, providing insights into local charge transfer dynamics and internal continuum states. We observe an acceleration of charge transfer times by almost one order of magnitude in thin-shell quantum dots, comprising only one or three double layers of ZnS, before reaching a size-independent limit. This size-dependence is governed by the existence of a faster charge transfer channel toward the CdSe core, only accessible for the inner-most shell layers, rather than a quantum confinement effect. By extending the traditional PCI model from free-electron systems to bound-state continua, we further establish a framework for interpreting line shape asymmetries and peak shifts that are frequently observed but often overlooked in resonant Auger measurements. We show that the strongly enhanced PCI in the samples with one or three double layers can be attributed to reduced collective electronic screening. This comprehensive experimental approach enables the simultaneous observation of collective electronic properties and atom-specific dynamics within in a single measurement under identical sample conditions, an advance particularly valuable for complex, sensitive materials.

## Introduction

The interplay of electron localization, itinerance, and charge transfer is essential to functional nanoparticles and quantum dots (QDs) [1–6]. In terms of electronic structure properties, materials on the nanoscale exhibit pronounced quantum confinement effects on their internal continua with a size-dependent transition from bulk-like band properties toward mixed and also atom-like localization [7]. Thus collective electronic states and local charge transfer between atomic units have significant impact.

Central properties of QDs such as absorption wavelength are collective properties of the entire system and are primarily determined by the particle size [8]. Advances in synthesis techniques have allowed for precise control over QD size, making these properties reliably tunable [9]. Other properties, such as photoluminescence quantum yield or RedOx chemistry, depend on a complex interplay of local and collective electronic structure aspects [10]. For example, quantum efficiency is strongly influenced by surface structure [11], the fabrication of core–shell and core–multishell architectures [12–13], and the choice of surface ligands [14].

**Figure 1:**
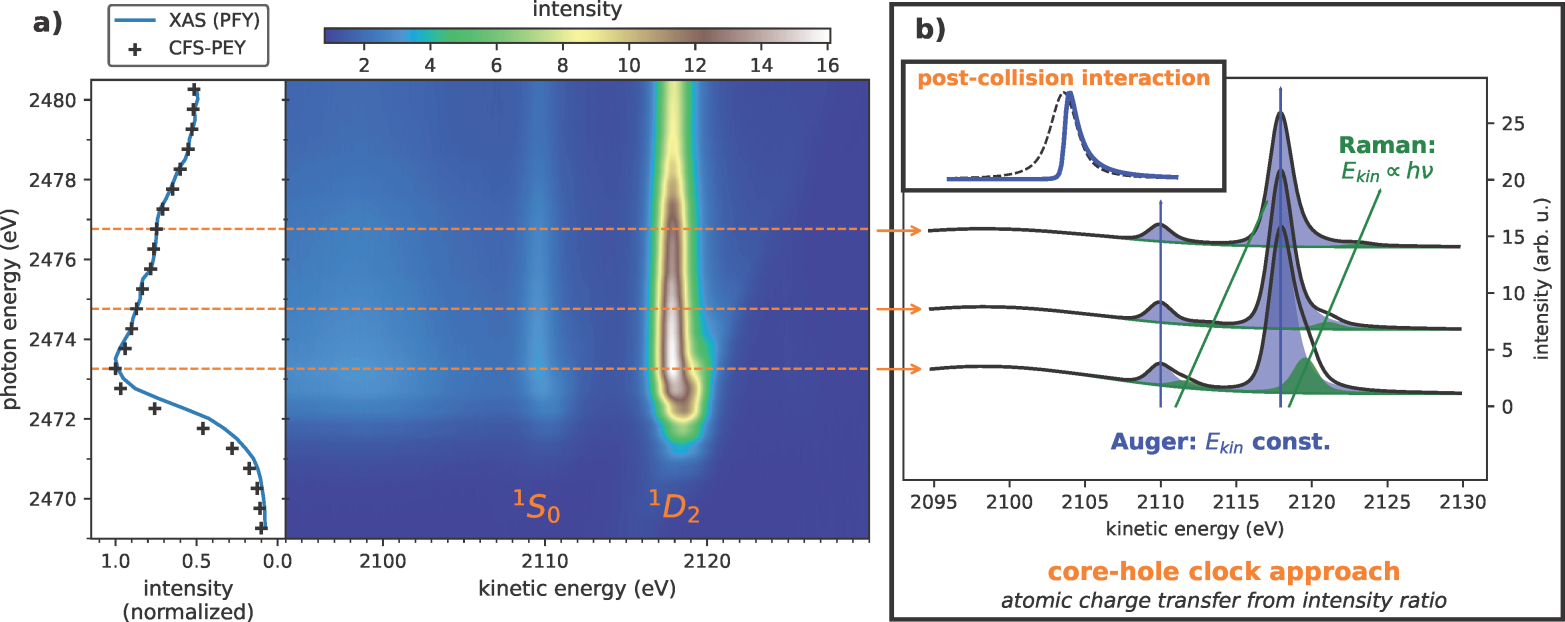
S KL_2_*_,_*_3_L_2_*_,_*_3_ resonant Auger measurements of core–shell nanoparticles (core: 3.5 nm CdSe, shell: 7 double layers ZnS): (a) Left: X-ray absorption spectrum in partial fluorescence yield (PFY, S Kα, blue solid line) and constant final-state partial electron yield (CFS-PEY, black markers). Right: Resonant Auger map containing the relevant S KL_2_*_,_*_3_L_2_*_,_*_3_ Auger multiplet. (b) Auger resonant Raman spectra at three photon energies (indicated in (a) as orange dashed lines). Each spectrum contains dispersionless normal Auger multiplet channels (blue), indicating charge transfer, and dispersed Raman Auger multiplet channels (green), reflecting atomic localization during the S 1s core-hole lifetime of 1.26 fs. The branching ratio relates charge transfer and core-hole life times. The atomic localized Raman Auger channels have a symmetric Voigt line shape. The Auger channels are asymmetric to higher kinetic energy. We assign this to post-collision interaction (PCI) via the internal charge transfer continuum within the nanoparticle. The inset highlights the asymmetric PCI line shape in comparison to the symmetric Voigt profile.

In this work, we obtain charge transfer on the atomic scale for size-dependent quantum-confined systems using core-hole clock (CHC) spectroscopy. We further expand the well-established CHC approach by the aspect of detecting the internal continuum states within the size-controlled nanoparticles via a line-shape analysis revealing post-collision interaction (PCI) effects within the conduction band continuum. We investigate CdSe/ZnS core–shell nanoparticles with a constant CdSe core size of 3.5 nm and varying shell thicknesses of ZnS that allow for atomic layer control with narrow size distribution. As indicated below in Figure 1, resonant Auger electron spectra are used to determine atomic charge transfer times based on the branching into localized and delocalized final states during the core-hole lifetime. We observe a significant reduction of charge transfer times within the ZnS shell for the thinnest shell samples. While the particles with one and three ZnS double layers show an up to one order of magnitude faster charge transfer, no variations are observed for the growing shells. We attribute that observation to the coexistence of an accelerated charge transfer channel toward the CdSe core, in addition to charge transfer happening solely within the shell, overshadowing possible small effects imposed by quantum confinement.

Also, the novel aspect of PCI involving the internal continuum of the respective size-controlled nanoparticle is reflected in the spectral line shape. Asymmetric line shapes and peak shifts are common to Auger resonant Raman scattering and resonant photoemission in atomic and molecular systems with free-electron continua and resonances leading to PCI. We propose that the general concept of PCI for free electrons can be extended and generalized to interacting electrons in bound-state internal continua of quantum-confined systems. Thus, the combination of PCI line shape analysis and core-hole clock spectroscopy not only allows one to derive atomically localized charge transfer times, but also the occurrence of internal continua defining collective properties of materials within a single measurement.

The experiment was done with freshly grown, commercially obtained CdSe/ZnS core–shell nanoparticles with a constant CdSe core size of 3.5 nm and varying shell thicknesses of 1, 3, 7, 11 and 15 double layers (DL) of ZnS (PlasmaChem GmbH). A full characterization of the QDs with UV–vis absorption spectroscopy, hard X-ray photoelectron spectroscopy (HAXPES), and near-edge X-ray absorption fine structure (NEXAFS) spectroscopy has been performed by us to verify the size-dependence of quantum confinement (see further details in Supporting Information File 1, sections 2–4). We then performed resonant S KL_2_*_,_*_3_L_2_*_,_*_3_ Auger spectroscopy with the core-hole clock approach [15] at the HIKE instrument at the BESSY II electron storage ring operated by Helmholtz-Zentrum Berlin für Materialien und Energie [16–17]. (Details of measurements and beamline given in Suppporting Information File Supporting Information File 1, section 1) In a single-electron picture, the S 1s core electron is resonantly excited into the unoccupied S 3p states. This S 1s^1^3p^+1^ core-excited state Auger-decays involving the S 2p electrons. If the resonantly excited electron undergoes charge transfer within the core-hole lifetime, a dispersionless S 1s^2^2p^4^3p^(deloc)^ final state is reached, whereas, without charge transfer, it results in a dispersing S 1s^2^2p^4^3p^+1^ final state, referred to as the resonant Raman Auger effect [18–19]. The branching ratio of these final states, as depicted below in Figure 1b, yields the charge transfer time in relation to the core-hole lifetime based on a model of independent exponential decay [20–21]. The validity and capabilities of the CHC approach was demonstrated across various sample systems, including gas adsorbates on surfaces [22–23], two-dimensional materials [24–26], nanoparticles [27], and matrix-embedded polymers [28].

## Results and Discussion

**Figure 2:**
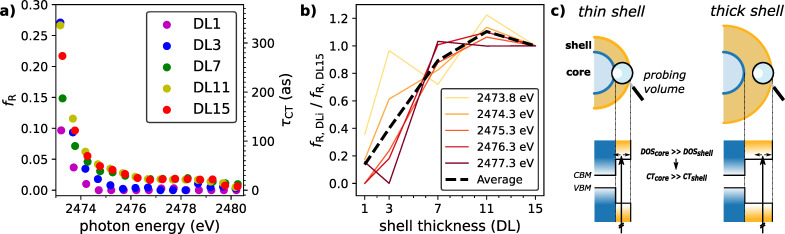
Charge transfer times of the core–shell nanoparticles (CdSe core diameter 3.5 nm) with different ZnS double-layer shell thicknesses (1, 2, 7, 11, 15 DL). (a) Raman fraction *f*_R_ and core-hole clock charge transfer times τ_CT_ from branching ratios of Auger and Raman channels as a function of excitation energy. Error bars for the highly constrained fit are within the size of the data points. (b) Size-dependence of charge transfer for all core–shell nanoparticles, each normalized to the largest (15 double layers) shell sample measured at five different photon energies. (c) The probing depth accentuates the outer over the inner contributions of the particles: Charge transfer within the ZnS shells is always detected. ZnS shell to CdSe-core charge transfer is also detected in thin-shell particles but to a lesser degree in thick-shell particles.

Figure 1a shows S KL_2_*_,_*_3_L_2_*_,_*_3_ Auger electron spectra at different photon energies around the absorption edge of S 1s electrons as a color-coded map. The intensity ratio between the Raman and Auger channels is obtained by fitting the resonant Auger spectra at the individual photon energies (see Supporting Information File 1, section 5, for detailed fitting procedure and model). Multiplying this ratio with the core-hole lifetime yields the charge transfer times presented in Figure 2a. To provide for a better comparison, relative charge transfer rates, each normalized to the charge transfer rate of the DL15 sample, at various photon energies are depicted in Figure 2b. Just above the resonance, these times are of the order of a few hundred attoseconds. For photon energies of 2475 eV and above, the charge transfer times are drastically reduced and converge to values below 10 as for the smallest quantum dots and to approximately 30 as for the largest quantum dots. These values are comparable to charge transfer times observed in PbS quantum dots [27] and other sulfur-containing nanomaterials [29,24].

The size dependence of the charge transfer times becomes particularly evident in the relative comparison. The charge transfer times increase by nearly one order of magnitude as the ZnS shell grows from one to seven double layers, whereas no significant differences are observed for shells thicker than seven double layers. The pronounced thickness dependence raises the question of the underlying mechanism responsible for the differences in the charge transfer times: Is the observed trend caused by changes in the quantum confinement, or does it arise from the coexistence of competing charge transfer channels?

Charge transfer times obtained from the core-hole clock approach probe the very local charge transfer to neighboring atomic sites and are particularly sensitive to the energetic and spatial overlap of the involved orbitals and the available density of states (DOS). Accordingly, they could be sensitive to changes in the DOS inflicted by an increased quantum confinement for the smallest shell QDs. In core–shell QDs, however, electron transfer can in principle occur either within the ZnS shell or toward the CdSe core, giving rise to two competing charge transfer channels with presumably different charge transfer rates. An energy level diagram of CdSe/ZnS QDs is schematically shown in Figure 2c. Owing to its smaller bandgap, the CdSe core is expected to have a higher density of states at a given energy compared to the wider-bandgap ZnS shell. From this perspective, charge transfer toward the core is expected to be faster than electron transfer within the shell. This implies that the charge delocalization is faster within those layers of the ZnS shell that are closest to the CdSe core.

Limited by the escape depth of the emitted electrons, the CHC approach is highly surface-sensitive. In the thinner QDs with only one or three double layers of ZnS, most of the probed volume consists of the first ZnS layer, which has the ability for rapid charge transfer toward the core. In the thicker-shell QDs, the first ZnS layer has an increasingly smaller contribution to the overall signal, resulting in the fact that only the slower charge transfer within the shell layers can be observed. At an electron kinetic energy of 2118 eV, the inelastic mean free path for ZnS is of the order of 4 nm [30]. Given a layer thickness of 0.3 nm for one ZnS shell layer [31], for the sample with seven double layers, already 40% of the Auger signal originates solely from the shell, while, in the shell with eleven double layers, this value increases to over 60% [32]. Given that the QDs are covered with a surface layer of long-chain hydrocarbon ligands (hexadecylamine) and that such films can reach film thicknesses of 1–2 nm [33], due to the additional attenuation, both values are still severely underestimating the contribution of the signal from the shell.

Given that an increasing quantum confinement for increasingly small shell thicknesses would results in a decreased density of states, one would expect a decreasing charge transfer rate, contrary to the observed acceleration. As further discussed in Supporting Information File 1, section 5, thin ZnS shells grow epitaxially to the CdSe core lattice, while thicker shells adopt the smaller unit size of bulk ZnS [34]. One would again expect shorter charge transfer times for larger shells due to the reduced unit cell size and thus an increased spatial overlap.

Summarizing the previous discussion, we attribute the observed size-dependent trends in the charge transfer times to the coexistence of two transfer channels, a fast channel toward the CdSe core and a slower transfer channel purely within the ZnS shell. Thus, in small shells, charge transfer times are significantly increased. Quantum confinement effects would be expected to show a contrary trend to our observations, indicating that the consequences of quantum confinement are likely small or negligible and that the overall size-dependence of the charge transfer is dominated by the competing channels within smallest shell systems, that is, systems under strong quantum confinement.

**Figure 3:**
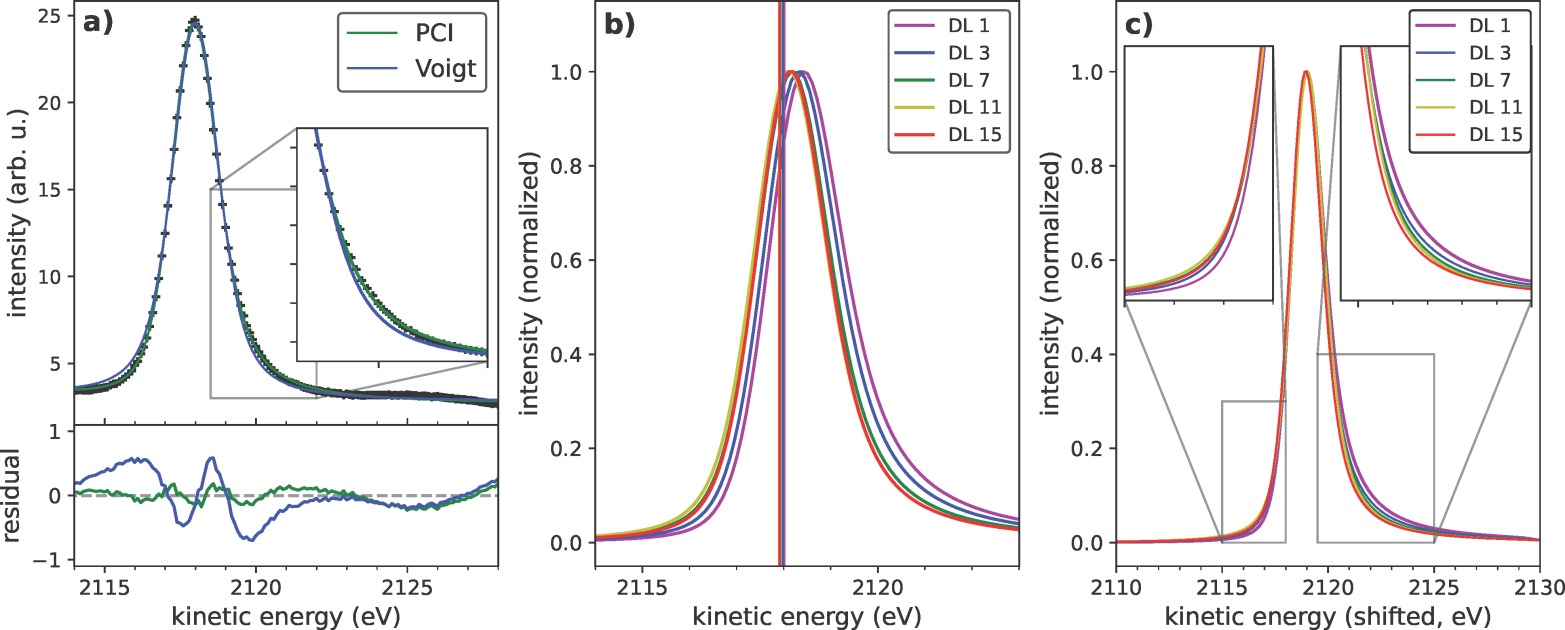
Post-collision interaction (PCI) between Auger and photoelectron interacting within the internal screening continuum of the nanoparticle. (a) The asymmetric PCI line shape describes the experimental line shape of the charge transfer channel significantly better than the symmetric Voigt profile. Integration of the resonant Auger spectra above the resonance yields a representative line shape for the Auger feature (black dots) as the influence of the dispersing Raman channel is suppressed. (b) Increasing PCI asymmetry with decreasing particle size, visible as an apparent peak shift. The unperturbed kinetic energies without the asymmetry induced by PCI, are shown as solid vertical lines for all nanoparticles and do not differ significantly. (c) With decreasing particle size, the asymmetry of the line shapes increases. Note that the spectra here are shifted to the same peak maximum positions to facilitate the comparison by eye.

The decomposition of the obtained resonant Auger spectra into the Auger and Raman components reveals an asymmetric Auger line towards higher kinetic energies. An average of the Auger component’s line shape is shown in Figure 3a. It is obtained by integrating the resonant spectra above the absorption edge between photon energies of 2474 and 2480 eV. Auger components are adding since they are at constant kinetic energy, whereas the Raman feature smears into a background due to its dispersion in photon energy. Effectively, this leads to a suppression of the contribution from the dispersing, low-intensity Raman feature and yields a high quality spectral shape of the photon-energy-independent Auger contribution. The deviation of this experimentally obtained Auger line shape from the classically expected Voigt profile becomes immediately evident within the shoulder regions of the peak. Additionally, even though the Auger channel is expected at constant kinetic energies, it appears in the RAS map (Figure 1a) to bend towards higher kinetic energies at photon energies close to the resonance. While asymmetries towards lower kinetic energies are typical for photoemission spectroscopy and are readily explained by various energy loss processes, this kind of behavior requires some process resulting in energy gain.

Typically, the emission of a photoelectron and the subsequent Auger electron emission are treated as separate and independent steps. However, a more elaborate description of the process is required to explain the observed line shapes, specifically considering the Coulomb interactions between the two electrons within the electric field of the ionized atom, creating a three-body problem. This is particularly pronounced for photoexciation above, but close to, the ionization threshold, where the high-energy Auger electron can overtake the slowly advancing photoelectron within the potential of the ion, subsequently gaining kinetic energy. These interactions, known as PCI effects, manifest in an apparent energy shift and increasing asymmetry of the Auger lines towards higher kinetic energies.

PCI effects were first observed by Barker and Berry in helium atoms [35]; since then, they have been widely studied in atoms, molecules, and gas-phase systems, leading to successful theoretical descriptions [36–37]. While these approaches allow one to derive the line shapes solely based on the kinematics of electron–electron interactions, the situation is more complex in condensed matter. The overall electron distribution can efficiently screen the ion charge, reducing the PCI effect [38]. Conversely, strong electron scattering reduces the effective attenuation length, slowing the photoelectron advancement; thus, it increases the PCI strength [39]. An increasing PCI effect is observed in growing multilayers of xenon on palladium due to decreasing screening from the metal surface [40], while increased screening decreases the strength of PCI effects on the La^3+^ Auger line in metallic LaB_6_ compared to insulating LaF_6_ [41].

For the fitting of the observed line shapes in this work, we utilized a model derived by Paripas et al. [42], based on the Eikonal approximation. The line shape *y*(ε) at relative energy ε is given by:


[1] $$y\left(\varepsilon \right)=y_{0}\frac{k\left(\xi ,\varepsilon \right)}{1+\varepsilon^{2}},$$

with *y*_0_ as a constant at a given interaction kinematics, an asymmetry term *k*(ξ, ε) depending on the asymmetry parameter ξ, and the relative energy ε = (*E* − *E*_0_)/0.5Γ_L_, where Γ_L_ is the natural linewidth, *E* the kinetic energy of the Auger electron, and *E*_0_ its unperturbed value (without PCI effects, details given in Supporting Information File 1, section 6). In Figure 3a, our model for the PCI line shape is applied to the previously obtained spectrum of the Auger feature. Comparing the residuals of the PCI line shape with those from a classical Voigt profile reveals a significantly enhanced fitting quality. Especially the characteristic asymmetries on both sides of the peak are well described by this model, emphasizing the appropriateness of the PCI approach. The asymmetry in the energy distribution of the Auger electrons, induced by PCI effects, also results in a shift of the peak maximum relative to the unperturbed Auger energy. These unperturbed values *E*_0_, derived from fitting the observed line shapes, are depicted as vertical lines in Figure 3b. Notably, while the peak maximum visibly shifts towards higher kinetic energies with decreasing quantum dot size, the fitted principal Auger energies remain nearly constant. This is expected since the HAXPES measurements show that the core level peaks of the differently sized quantum dots do not exhibit any significant variations in binding energy within the experimental resolution (Supporting Information File 1, section 3). Therefore, the observed peak shift is primarily attributed to variations in the asymmetry of the line shape and, thus, linked to the strength of the PCI effect.

**Figure 4:**
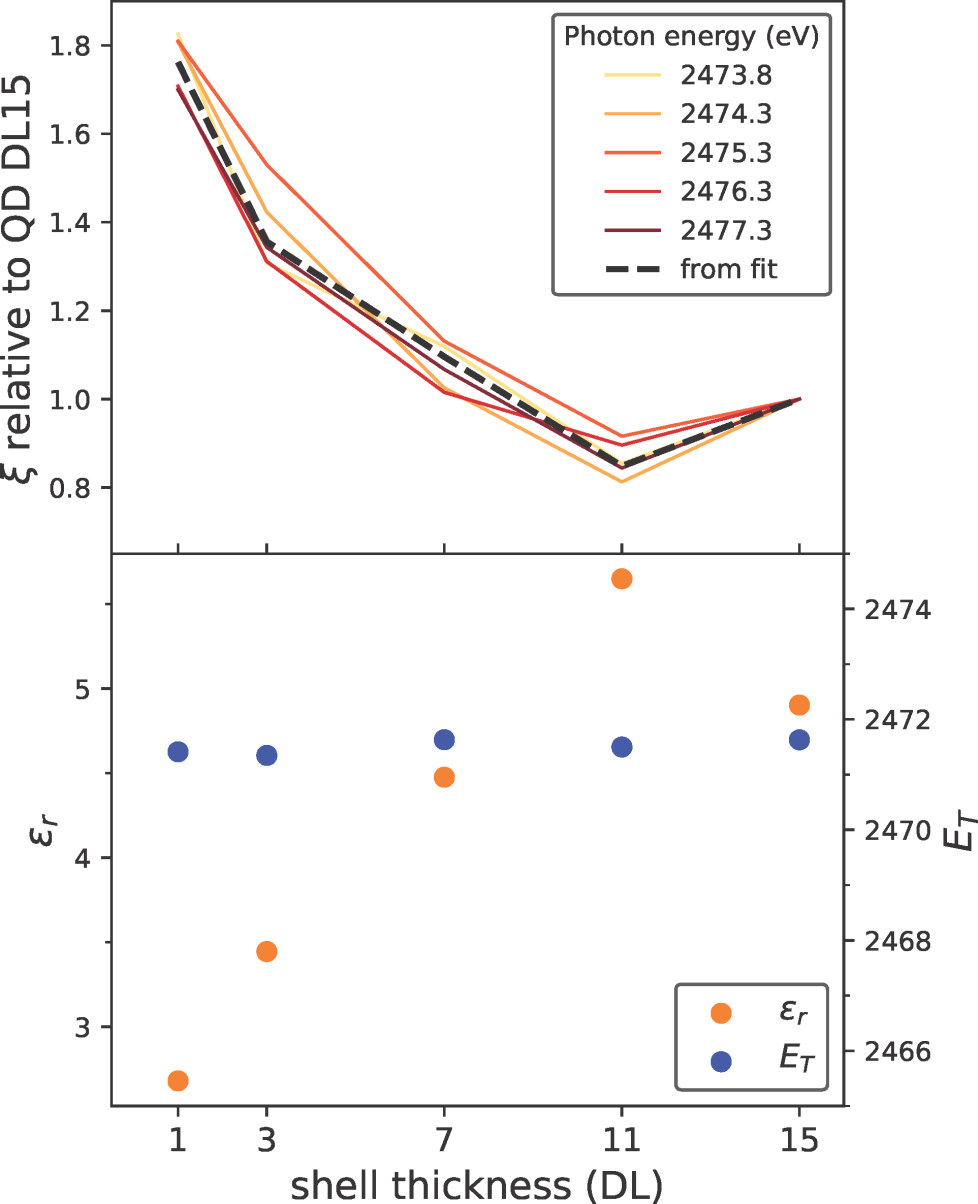
Size-dependence of the PCI asymmetry parameter. Top panel: Asymmetry parameter ξ for nanoparticles with different shell thicknesses, each normalized to the asymmetry parameter of the biggest nanoparticle (15 double layers of ZnS) at different photon energies. Bottom panel: Threshold energies *E*_T_ and relative permittivities ε_r_ obtained from fitting the asymmetry parameters with our PCI model as a function of particle size.

The magnitude of the PCI effect, and consequently its impact on the line shape, is ultimately governed by the asymmetry parameter ξ, shown normalized to the asymmetry parameter of the DL15 sample in Figure 4 (top panel) at various photon energies as a function of particle size. It is clear that the asymmetry parameters increase for smaller QDs. The consistency of this trend across different photon energies strongly suggests that the observed variations are indeed the result of a size-dependent process.

To gain further insight into the PCI effect, we modeled the observed evolution of the asymmetry parameter as a function of the particle size. As previously discussed, the strength of PCI in condensed matter is determined by the interaction kinematics, the system’s screening capabilities, and the degree of localization of the outgoing photoelectron. Within the framework of the employed Eikonal approximation, the interaction between the two electrons within a vacuum continuum is determined by the velocities of the Auger electron 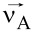 and photoelectron 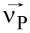 as 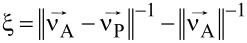 [42]. To determine the velocities, we used the previously obtained unperturbed Auger kinetic energy *E*_0_ for the Auger electron and a kinetic energy *h*ν − *E*_T_ for the excited electron, with an initially arbitrary threshold energy *E*_T_. While in typical above-ionization-threshold excitations, this threshold energy corresponds to the ionization potential, our case involves resonant excitation where the electron remains in bound states. To account for the propagation of the electrons within the crystal lattice and screening effects, we additionally included the effective masses and the dielectric constant ε_r_ into our model [39], resulting in the following model for the asymmetry parameter ξ (details are given in Supporting Information File 1, section 6):


[2] $$\xi =\frac{\sqrt{m_{\text{e}}^{*}}}{\sqrt{2}\varepsilon_{\text{r}}}\left(\frac{1}{\sqrt{h\nu -E_{\text{T}}}-\sqrt{E_{0}}}-\frac{1}{\sqrt{E_{0}}}\right).$$

**Figure 5:**
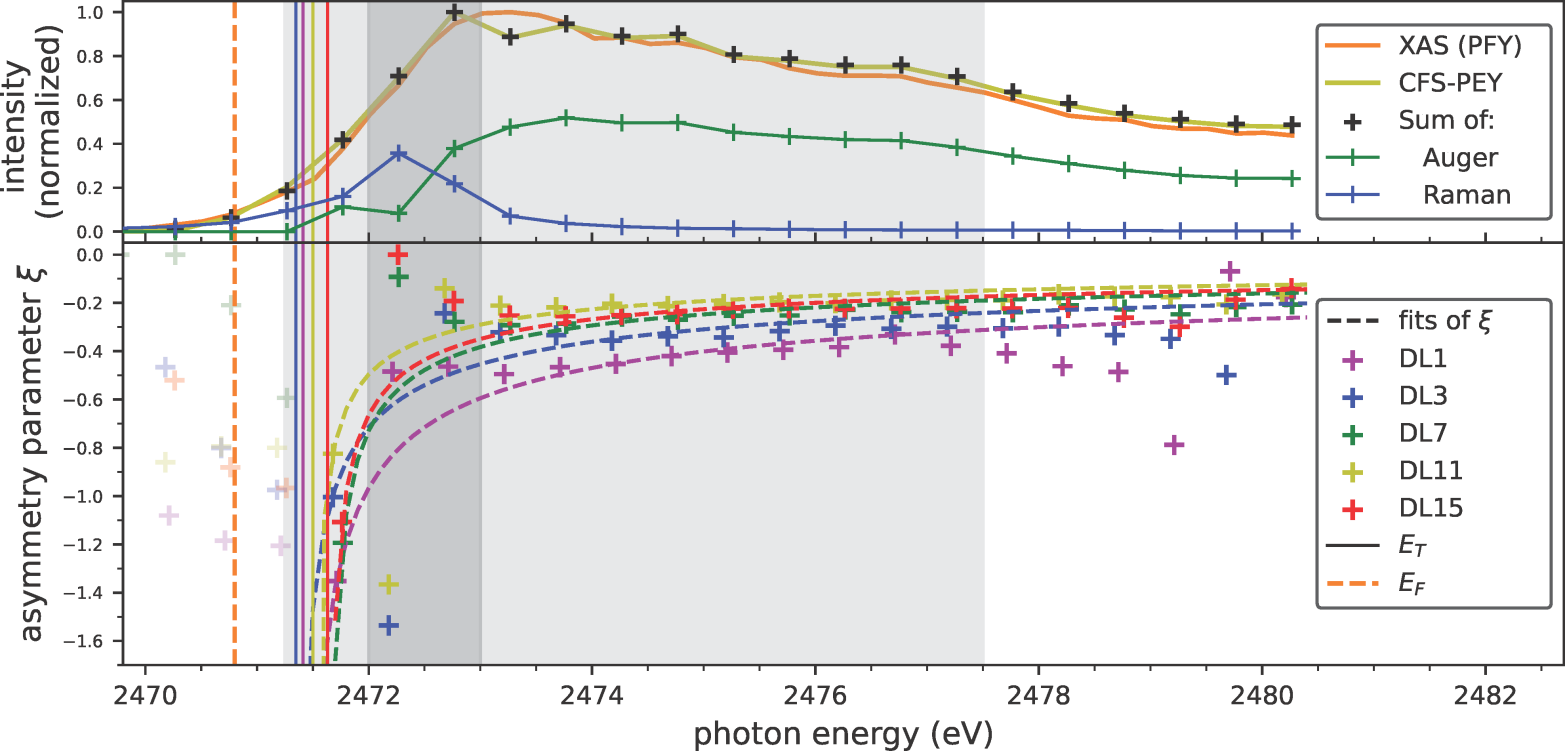
Variation of internal charge transfer continuum coupling as seen by the PCI parameter as a function of photon energy and particle size. Top panel: The X-ray absorption cross section (PFY, orange line) is shown in comparison to an integration of the resonant Auger spectra (CFS-PEY, light green line). Also, the deconvolution of the signal into the intensities of Auger (green) and Raman (blue) channels, as well as their sum (black crosses), are shown. Bottom panel: Comparison of the asymmetry parameter ξ for the differently sized QDs as a function of photon energy around the S K-edge. Fits of the evolution of the asymmetry parameters according to our adjusted PCI model are shown as dashed lines. In both panels, an estimation of the Fermi level (dashed orange line) and the fitted threshold energies *E*_T_ from the PCI model (solid vertical line) are shown. The light gray area corresponds to a continuum situation, which is, according to our interpretation, the conduction band. The dark gray area is governed by the S 3p atomic resonance.

As shown in Figure 4 (top panel, black dashed line) and Figure 5 (bottom panel, dashed lines), this model fits the data remarkably well, particularly considering the model’s simplicity and the complexity of extracting the asymmetry parameters from deconvoluted resonant Auger spectra. The unperturbed Auger kinetic energies *E*_0_, shown in Figure 3b (vertical lines), as well as the fitted threshold energies *E*_T_, shown in Figure 4 (bottom panel), are consistent across the various samples. HAXPES measurements also show no significant changes in electronic structure. Therefore, the differences in observed asymmetries are attributed to variations in screening capability. The resulting values for the dielectric constant ε_r_ are displayed in Figure 4 (bottom panel), exhibiting a trend matching the evolution of the relative asymmetry parameters. As expected, increasing shell thickness enhances electron density, leading to a higher dielectric constant and improved screening ability. These findings and the absolute ε_r_ values align with theoretical predictions for nanoparticles in general and observations on ZnS nanomaterials [43–45].

By combining all previously discussed results in Figure 5, a comprehensive understanding of the electronic processes following resonant excitation in quantum dots is achieved. The S 1s binding energy, obtained via normal photoemission spectroscopy relative to the Fermi level, allows for an estimation of the Fermi level, represented as a horizontal orange line in Figure 5. According to our interpretation, the threshold energies *E*_T_, derived from fitting the PCI asymmetry parameters ξ and shown as solid vertical lines, correspond to the conduction band minima (CBM). This interpretation is compatible with electronic structure calculations of ZnS nanoparticles, according to which the Fermi level is approximately 0.4 eV below the CBM [46]; we obtained a similar value of approximately 0.7 eV.

Although PCI effects are typically associated with excitations into the vacuum continuum, internal continua equally yield identical processes. In our case, the core-excited electron residing in the continuum Bloch states of the unoccupied conduction band can vary its energy within this internal continuum. By allowing the excited electron to delocalize and leave the excitation site, similar PCI effects as in the vacuum continuum will take place. While discrete energy states would only lead to the observation of particular satellite features, only a continuum provides a sufficient density of states to allow for arbitrary energy sharing and, thus, the continuous deformation of the Auger line. With respect to the PCI line shape, this differentiates the conduction band continuum from the valence band continuum, which is occupied in our semiconducting nanoparticles and, thus, does not allow for energy redistribution within itself.

In fact, examining the evolution of the charge transfer times and of the asymmetry parameter across photon energies above the proposed CBM (light gray area in Figure 5), except within the dark gray area, both follow expectations consistent with a continuum situation. For the asymmetry parameter, this is especially evident due to the remarkable agreement between experimentally derived parameters (colored crosses) and the trend from the adapted PCI model for continua cases (colored dashed lines). Note that we have excluded the dark gray region from the analysis and the fit with the PCI model due to the unresolved nature of this feature. We discuss this aspect in depth in the following paragraph. While PCI effects have previously been investigated primarily for above-ionization cases, PCI-like effects in bound states have been either ignored [39] or described by new kinds of processes [28]. Here, we demonstrate that the PCI process is indeed a general effect in continua, governed by the same physical principles and models regardless of whether the continuum is the vacuum or the conduction band.

Within the dark gray area, the asymmetry appears to vanish and drastically deviates from the PCI trend. Based on the photon energy, this region corresponds to excitations into the atomic S 3p resonance. The localized nature of this resonance becomes further evident by the significantly enhanced intensity of the Raman feature (Figure 5, top panel, blue line) and significantly increased charge transfer times. While an underestimation of the asymmetry in this region could be attributed to inadequate fitting, since the Raman and Auger features completely overlap within this area, we argue that the atomic resonance is not a suitable continuum state to allow for PCI. Being a highly localized state, it is rather the opposite of a continuum and, hence, does not provide a sufficient number of energy states that are needed for the continuous energy sharing between the electrons in a PCI process.

Another interesting observation is the difference between the pronounced fluctuations between the relative charge transfer times (Figure 2b) and the smooth trend of the relative asymmetry factors (Figure 4, top). While the asymmetry and the PCI effect are mostly governed by the screening and, thus, the overall electron density of the nanoparticle, the charge transfer times depend on the very local structure of the nearest atomic neighbors. As the shell growth of CdSe/ZnS QDs is known to be highly anisotropic and often exhibits crystallinity issues due to the lattice mismatch between CdSe and ZnS in zinc blende or wurtzite lattices [12,47], the relatively high fluctuations of the charge transfer times seem reasonable. In comparison, the electron density can be seen more as a collective property depending on the size of the particles and is, thus, much less sensitive to structural impurities or variations.

## Conclusion

The design of new high-performance devices necessitates an understanding and control of both collective properties and characteristics that are highly sensitive to local atomic changes. Our approach allows for the observation and measurement of both types of effects within a single sample under identical conditions, thereby significantly enhancing the investigation of complex sample systems that may be susceptible to structural changes when prepared or studied using different experimental techniques. However, due to the multitude of processes and contributing factors, distinguishing and fully understanding them requires sophisticated analysis and interpretation. In this work, we present an experimental approach based on resonant Auger spectroscopy, which not only enables element-specific and resonantly enhanced spectroscopy, but also provides sensitivity to atomically localized changes and collective properties within one measurement. While the atomic charge transfer times are obtained using the well-established core-hole clock approach, we generalized the theory of post-collision interaction from a vacuum continuum situation toward bound-state continua, thus creating a probe for the overall electron screening. Employing this approach to CdSe/ZnS core–shell quantum dots, we observe a size-dependence for both the local charge transfer and the collective electron screening.

We observe a significant decrease of the charge transfer times about one order of magnitude for the thinnest shell quantum dots with only one or three double layers of ZnS. This acceleration of charge transfer is attributed to the presence of an additional, faster electron delocalization channel toward the CdSe core. Based on the band alignment in the core–shell system and the theory of quantum confinement, we argue that the CdSe core possesses an increased density of states and, thus, allows for faster charge transfer. As charge transfer toward the core is only possible for the first shell layers, the observed acceleration of the charge delocalization cannot be attributed to a change in quantum confinement.

We have shown that the well-studied concept of post-collision interaction can be successfully used to explain asymmetries and peak shifts for excitations into bound continuum states within the conduction band. We use the fact that the theoretical framework of PCI requires a continuum of energy states in which the electrons have sufficient available states to gain or loose variable amounts of energy, which makes a continuous shift of the energy distribution possible. The fully established theoretical description of PCI in a vacuum continuum can be applied to a bound continuum like the conduction band by modifying the vacuum model of PCI through the introduction of the dielectric constant to account for the screening within the solid. For the quantum dot system, we observe a significant increase of the dielectric constant with growing shell thickness, attributed to an increased electronic density. Thus, in contrast to the size-dependence of the atomically localized charge transfer, the collective property of electron density is severely affected by quantum confinement.

Our findings expand the application of resonant Auger measurements in general, and we show, for size-controlled core–shell quantum dots, how to obtain previously inaccessible data of local charge transfer and collective dielectric response. The PCI core-hole clock analysis is, thus, a synchrotron approach of general relevance to the characterization of materials involving bound-state continua.

## Supporting Information

File 1 Experimental details, sample characterization (UV–vis/HAXPES/NEXAFS), fitting procedure and details about PCI line shape not included in the main text.

## Data Availability

Data generated and analyzed during this study is available from the corresponding author upon reasonable request.
