# Supplementary material for: Probing internal continua and atomic ultrafast charge transfer within size-controlled nanoparticles by post-collision interaction in core-hole clock spectroscopy
Source: Beilstein J Nanotechnol. 2026 Apr 7;17:505–14. doi: 10.3762/bjnano.17.33 (PMC13071936; doi:10.3762/bjnano.17.33)
Supplement: File 1 — Experimental details, sample characterization (UV–vis/HAXPES/NEXAFS), fitting procedure and details about PCI line shape not included in the main text. [file Beilstein_J_Nanotechnol-17-505-s001.pdf]

BEILSTEIN JOURNAL OF NANOTECHNOLOGY

Supporting Information

for

Probing internal continua and atomic ultrafast charge transfer within size-controlled nanoparticles by post-collision interaction in core-hole clock spectroscopy

Johannes Lütgert, Erika Giangrisostomi, Nomi L. A. N. Sorgenfrei and Alexander Föhlisch

Beilstein J. Nanotechnol. **2026,** *17,* 505–514. doi:10.3762/bjnano.17.33

Experimental details, sample characterization (UV–vis/ HAXPES/NEXAFS), fitting procedure and details about PCI line shape not included in the main text

License and Terms: This is a supporting information file under the terms of the Creative Commons Attribution License (https://creativecommons.org/licenses/by/4.0). Please note that the reuse, redistribution and reproduction in particular requires that the author(s) and source are credited and that individual graphics may be subject to special legal provisions.
The license is subject to the *Beilstein Journal of Nanotechnology* terms and conditions: (https://www.beilstein-journals.org/bjnano/terms)

1 Experimental Details

1.1 Samples and materials

CdSe/ZnS core–shell nanoparticles with varying shell thicknesses of 1, 3, 7, 11, and 15 double layers of ZnS were obtained from PlasmaChem GmbH. The preparation includes the growth of bare CdSe cores with an approximate diameter of 3.5 nm in the first step and a gradual growth of the ZnS shell, thus assuring the same core size for all samples. Solvents, namely toluene (Macron Fine Chemicals, purity ≥ 99.5 %), chloroform (Fisher Scientific, purity ≥ 99 %) and ethanol (Sigma-Aldrich, purity ≥ 99.8 %) were commercially obtained. The sample substrate for all synchrotron experiments was indium tin oxide (ITO)-coated quartz glass with a surface resistivity of 8–12 Ω commercially obtained from Sigma-Aldrich.

1.2 Sample preparation for X-ray experiments

Roughly 10 mg of the respective nanoparticle sample were dissolved in 500 µL toluene and treated in an ultrasonicator until there was no more recognizable solid residual, yielding strongly red, but colloidal and cloudy, solutions. Transparent solutions were obtained by subsequent filtering through syringe filters with a pore size of 200 nm. The substrates, ITO on quartz glass, were thoroughly cleaned with ethanol before spin-coating and then quickly mounted onto the spin-coater. Spin-coating was done at a rotational speed of 2000 rpm for 30 s. As soon as the desired speed was reached, 30 µL of the previously prepared nanoparticle solutions were quickly deposited in the middle of the spinning substrate. To achieve the desired film thickness, the deposition procedure was repeated four times. The finished samples were obtained as slightly red thin films. Substrates were mounted on tantalum sample holders with carbon tape and quickly transferred into the ultrahigh vacuum system of the experimental station to prevent surface oxidation or contamination.

1.3 X-ray experiments

All X-ray absorption and photoelectron experiments were performed at the HIKE experimental station [1] at the KMC-1 beamline [2] at the electron storage ring BESSY II operated by Helmholtz-

Zentrum Berlin. The dipole beamline provides linear-horizontal polarized X-ray radiation with photon energies between 2 and 12 keV. In the photon energy range between 2.4 and 3 keV used in this work, the X-ray flux was of the order of 1 10^{11} ph $*$ s^{-1} with an energy resolution between 200 and 300 meV. An ionization chamber filled with nitrogen gas at a pressure of 2 mbar within the beam path was used to constantly monitor the photon flux.

All experiments were performed under ultrahigh-vacuum conditions with pressures of the order of 10^{-8} mbar. Photoemission experiments were performed using a VG Scienta R4000 hemispherical electron analyzer oriented at an angle of 90 ° with respect to the photon beam. A Bruker XFlash 4010 fluorescence detector at an angle of 45 ° to the X-ray beam was used for NEXAFS measurements.

NEXAFS measurements were performed in a photon energy range between 2450 and 2560 eV with a step size of 0.25 eV. The partial fluorescence yield was obtained by measuring the fluorescence signal from the sulfur Kα decay with an energy window of 87 % around the emission line. The photon beam was impinging at an angle of 45 ° relative to the sample, and the fluorescence detector was in normal orientation to the sample surface. The photon flux was constantly measured using the ionization current of the ionization chamber and the XAS spectra were normalized to the flux during the data analysis.

For photoemission experiments, the X-ray beam was impinging at a gracing incident angle of 9 °, while the detector was roughly perpendicular to the sample surface at an angle of 81 °. Survey spectra were recorded with a photon energy of 3000 eV in a kinetic energy range between 400 and 3005 eV using a pass energy of 500 eV and energy steps of 0.5 eV. Resonant Auger measurements were conducted with a pass energy of 200 eV and energy steps of 0.1 eV. Photon energies were varied between 2474 and 2485 eV in 0.5 eV steps. For each photon energy, a spectrum of the S KLL Auger lines was measured at kinetic energies between 2090 and 2125 eV, as well as a spectrum of the Zn $2p_{3/2}$ photoline as a reference spectrum. The latter was measured in a kinetic energy range of 1438–1453 eV at a photon energy of 2474 eV, and the range was gradually increased by 0.5 eV for every step of increasing photon energy.

1.4 Data analysis

Data analysis was performed with Python, version 3.12.0, using the packages “NumPy” for data handling [3], “SciPy” for numerical mathematical operations [4] and “Matplotlib” for data visualization [5]. Fitting was done using non-linear least-square minimization algorithms provided by the “lmfit” package [6]. If not otherwise stated in the text, pre-built fitting functions from “lmfit” were used. For all fits of photoemission spectra, the uncertainties given by the square root of the measured intensity according to Poisson statistics were used as a weight for the data points.

A calibration of the electron kinetic energy was performed by regularly measuring the photoelectron spectrum of the Au 4f core levels of a gold foil using photon energies of 3000 eV for survey spectra and 2475 eV for resonant Auger measurements. Due to an insufficient reproducibility of the beamline photon energy, the surveys were further calibrated. For the resonant Auger measurements, the Zn $2p_{3/2}$ reference spectra were fitted, and the relative offsets between the fitted binding energies for different photon energies were used to calibrate the kinetic energy of the Auger spectra. The total integrated intensity of each Zn $2p_{3/2}$ spectrum was used to normalize the intensity of the corresponding Auger spectrum.

1.5 UV–vis absorption measurements

The nanoparticle samples were dissolved in toluene. The solution was treated in an ultrasonicator until all visible solid residuals were dissolved, yielding slightly cloudy solutions. Subsequent filtration using syringe filters with pore sizes of 200 nm resulted in clear and transparent solutions used for the absorption measurements. The solutions were individually diluted so that a suitable transmission could be achieved. As the bare CdSe core sample was not treated with surface ligands, it was almost completely insoluble in toluene. Accordingly, more polar chloroform was used as a solvent. Measurements of the core sample were done on a highly colloidal solution as attempts to filter the solution completely depleted the signal.

UV–vis measurements were performed on a Agilent Cary 5000 Series UV–vis-NIR Spectrophotome-

ter at wavelengths between 200 and 1100 nm. A pure solution of toluene or chloroform was used for the reference measurement and baseline correction.

2 UV–vis Absorption Spectroscopy

An increasing bandgap, manifested as a decreasing absorption wavelength, is one of the most prominent indicators for increasing quantum confinement effects induced by a decrease in the size of the confined system. Such a trend is clearly visible in the absorption spectra (Figure S1) of the studied nanoparticle samples. The well-established empirical equation by Yu et. al. [7] correlates the absorption wavelength of the first absorption feature (**A**) with the particle size for CdSe nanoparticles. The diameter of the CdSe cores in the present samples can be determined to be 3.45 nm. Even though the precise shell thickness cannot be determined using UV–vis spectroscopy, the trend of growing shells is clearly reflected in the data as a decreasing quantum confinement.

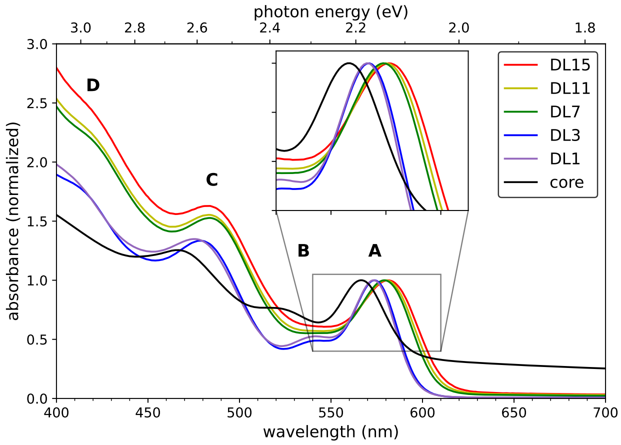

Figure S1: Size-dependence of the UV–vis absorption spectra.

Using Tauc's method, the bandgap can be determined directly from the absorption spectra. The approach is shown in Figure S2a, with the resulting bandgaps in Figure S2b. The aforementioned trend of decreasing quantum confinement with growing nanoparticle size is again clearly visible.

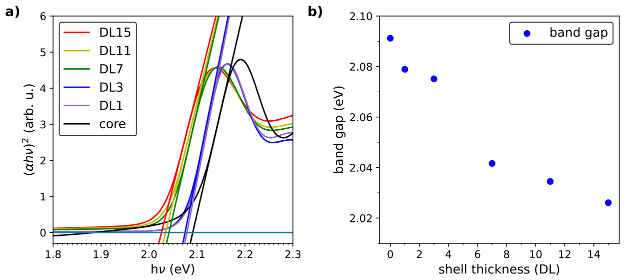

Figure S2: a) Determination of bandgaps using Tauc's method. b) Size-dependence of the bandgap for different shell thicknesses.

3 Hard X-Ray Photoemission Spectroscopy (HAXPES)

Survey spectra for the differently sized nanoparticles are shown in Figure S3. They confirm the expected composition of the CdSe/ZnS core–shell nanoparticles. The carbon C 1s signal is explained by the capping layer of long-chain organic ligands, mainly hexadecyl amine $C_{16}H_{35}N$. Due to the large probing depth achieved with 3000 eV X-rays and an inhomogeneous surface coverage, the silicon Si 1s and oxygen O 1s signals are attributed to the quartz glass (SiO_2) substrate. We expect the phosphorus P 1s signal to originate from residual phosphor-containing solvents like trioctylphosphine (TOP) or trioctylphosphine oxide (TOPO), which are commonly used as solvents in the high-temperature synthesis of nanoparticles and are reported as common impurities [8-10].

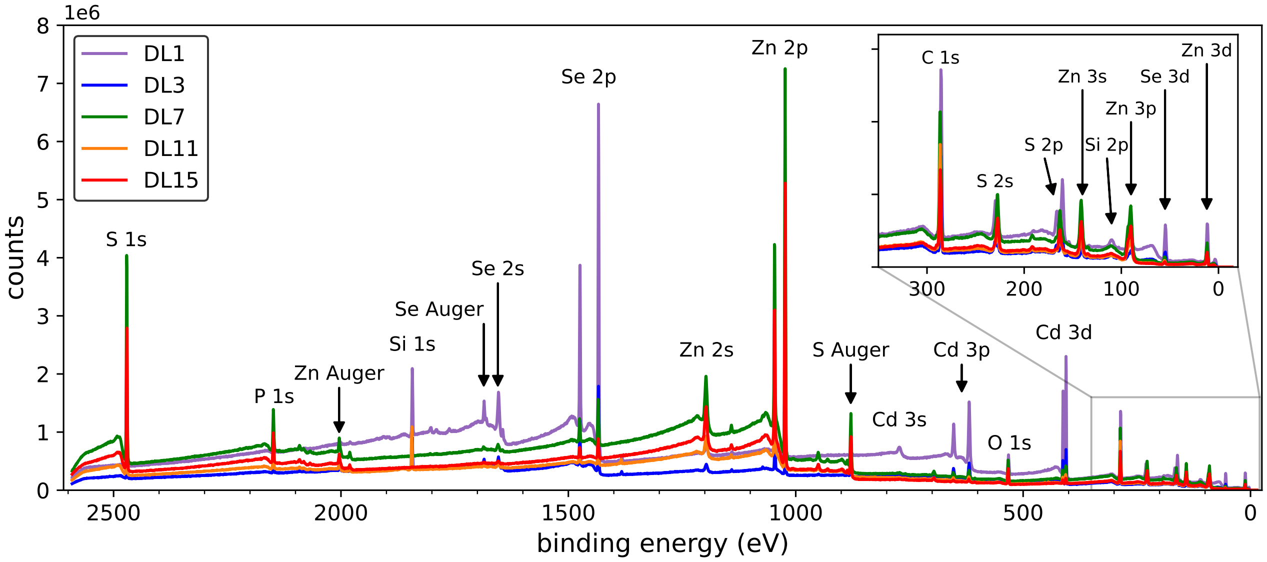

Figure S3: HAXPES survey spectra of the differently sized nanoparticles obtained with a photon energy of 3000 eV.

For all relevant chemical elements in the nanoparticles, namely cadmium, selenium, zinc and sulfur, the core levels shown in Figure S4 do not show any differences in the peak shape or binding energy within the available energy resolution. Comparing the binding energies of the nanoparticles Zn $2p_{3/2}$ and Cd $3d_{5/2}$ lines with the respective values for bulk ZnS [11] and bulk CdSe [12] (blue dashed lines), the binding energy increases for the nanoparticles by 1.5 and 1.0 eV, respectively. Thus we

observe a quantum confinement effect on the core level electronic structure, but it is not changing significantly for the different shell thicknesses investigated within this work. This is in accordance with literature reports on other nanoparticle systems [13].

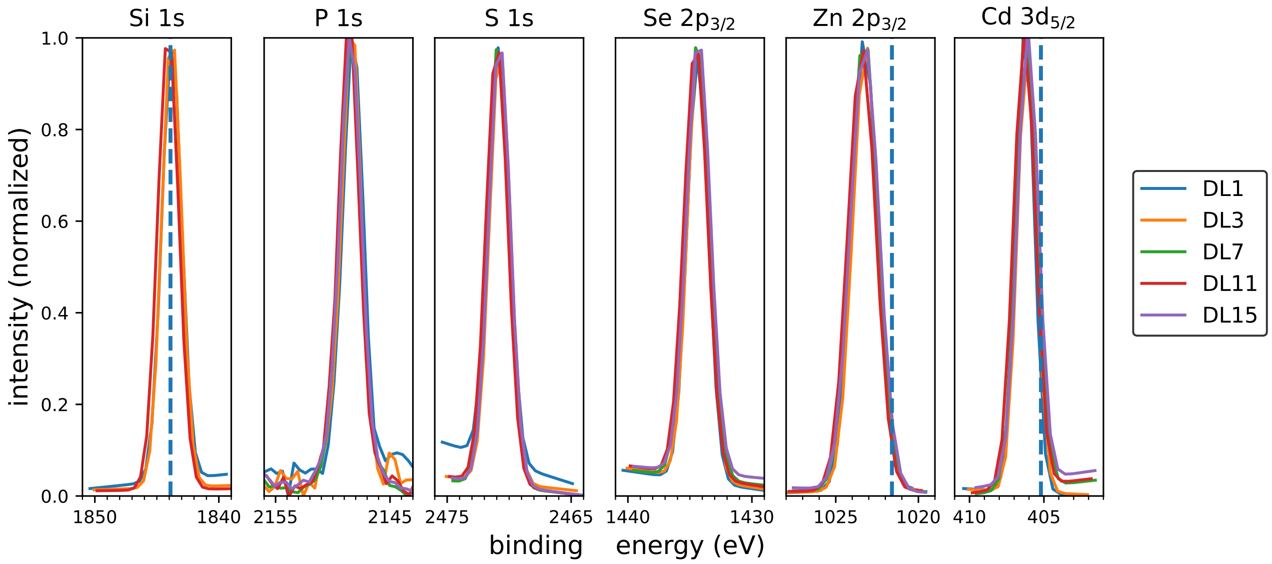

Figure S4: Selected core level spectra for the differently sized nanoparticle samples obtained from HAXPES, from left to right: Si 1*s*, P 1*s*, S 1*s*, Se $2p_{3/2}$, Zn $2p_{3/2}$, and Cd $3d_{5/2}$. Literature reference spectra are shown as blue dashed lines for quartz glass (Si 1*s*), bulk ZnS (Zn $2p_{3/2}$), and bulk CdSe (Cd $3d_{5/2}$). Note that, as an energy calibration, the Si 1s line is set to the reported binding energy of 1843.8 eV [14] for Si 1s in quartz glass (SiO_2) as the substrate's electronic structure should be independent of the respective nanoparticle dimensions. As the samples with seven and fifteen double layers do not show a Si 1s signal, they were corrected using the P 1s photoline, which was observed at a consistent binding energy for the other nanoparticles.

4 Near-Edge X-Ray Absorption Fine Structure (NEXAFS)

The NEXAFS spectra around the S K-edge at 2473 eV, corresponding to transitions from S 1s core levels into unoccupied S 3p states of S^{2-} ions, are shown in Figure S5 for the differently sized nanoparticles. Compared to the binding energy of the sulfur S 1s level of 2470.8 eV obtained from photoemission spectroscopy, the primary unoccupied state responsible for the absorption edge lies 2.2 eV above the Fermi level. While the photon energy of the absorption edge itself does not change between the different shell thicknesses, the near edge region of the smallest shells with one and three double layers of ZnS differs significantly from the spectra of the other sizes.

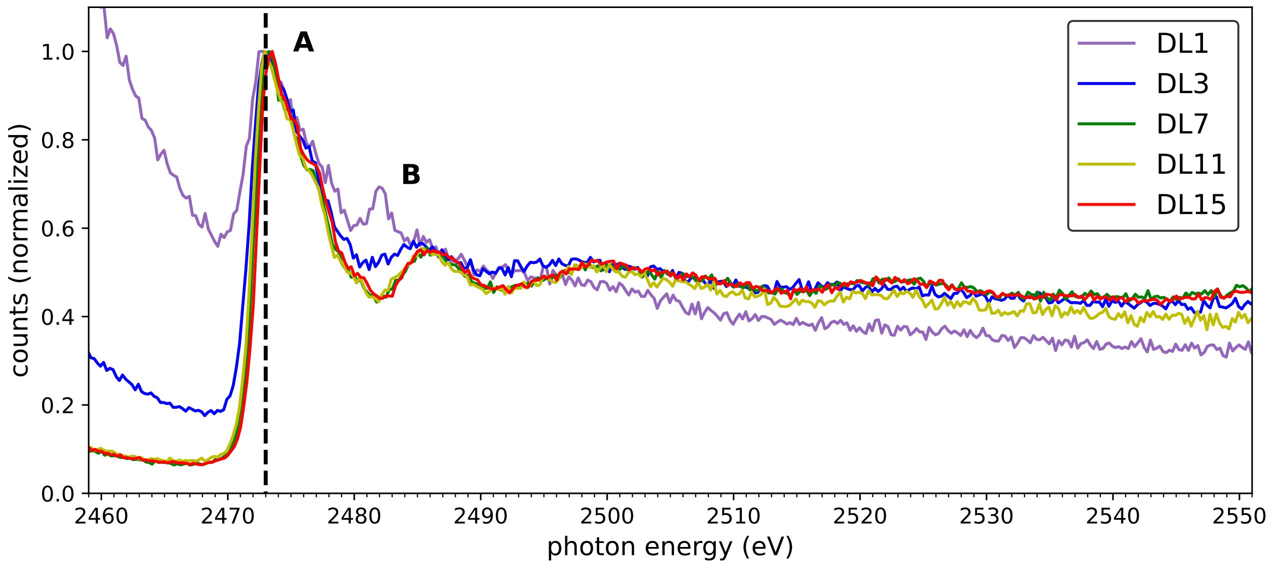

Figure S5: S K-edge NEXAFS spectra normalized to the absorption edge maximum for the differently sized nanoparticles.

While feature **A** and its fine structure are similar to reported data for ZnS in a wurtzite or zinc blende structure [15,16], feature **B** is the result of multiple scattering, and its energy position depends on the lattice constant [16]. The ZnS shell in CdSe/ZnS core–shell nanoparticles was proven to adopt the same crystal structure as bulk ZnS, even though the lattice constants differ significantly by roughly 12 % [17]. It is reported that, for thin shells up to two double layers, the ZnS shell preferentially grows epitaxially to the core lattice, while for thicker shells it adopts the smaller lattice constant of bulk ZnS introducing defects in the crystal [17]. Accordingly, no differences in the local structure are expected

for the samples with shell thicknesses above three double layers, and their multiple scattering regions are indeed unchanged. For the smallest sample with just one double layer, an epitaxial growth with a larger lattice constant is expected and is indeed observed as a shifted multiple scattering peak **B** towards lower photon energies [16]. An intermediate situation seems reasonable for the three-double-layer shell. As the shell growth is proven to be highly anisotropic and inhomogeneous [17], it can be expected that this sample contains areas with two or fewer, as well as areas with more than two, double layers.

It should be noted that no additional features are visible. In particular, there are no signs of the formation of CdS, indicating a well-separated interface between core and shell. For instance in InP/ZnS quantum dots, a penetration of sulfur ions into the core is experimentally observed in the NEXAFS spectra [18].

5 Fitting Procedure for S $KL_{2,3}L_{2,3}$ Resonant Spectra

Figure S6 illustrates for an exemplary spectrum the components of the model used to fit the resonant Auger spectra. Due to the complexity and large number of parameters of this model, we decided for a highly constrained fitting approach.

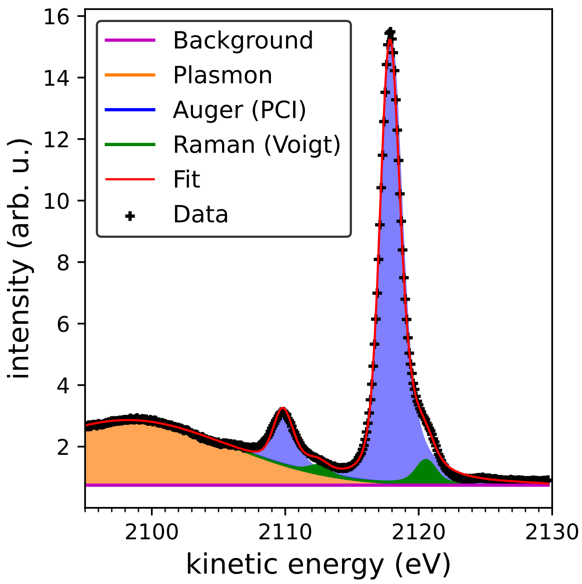

Figure S6: Components of fitting model for exemplary resonant Auger spectrum.

The background is described by a combination of a linear function and a Gaussian profile to model the broad feature, probably a plasmon loss feature, on the low-kinetic-energy side. The signals of interest consist of a PCI line shape (implemented according to section 6) for the delocalized Auger channel and a Voigt profile for the localized Raman channel. Both components are included twice, one for the $^1S_0$ and another for the $^1D_2$ multiplet component.

The spectra for all photon energies are iteratively fitted. The parameters for the background are fixed to the best values for every photon energy already early on in the iterations. The instrumental broadening σ included in both the PCI line shape and the Gaussian contribution to the Voigt profile is fixed to a value of 0.53 eV obtained from fitting the Zn $2p_{3/2}$ core level with Voigt profiles for all photon energies. The Lorentzian contribution to the Voigt profile and the linewidth of the PCI line shape Γ_L are set to the same optimal value for all signals. The unperturbed energy position E_0 of the Auger-PCI line shapes does not show significant trends at varying photon energies and, accordingly,

is fixed. The energy position of the Raman-Voigt profil is set to linearly disperse with the photon energy, while the starting value $E_{R,0}$ is fixed. The intensity ratio between the Auger and Raman signals is fixed for both components of the multiplet. In the final iteration, the only non-constrained parameters are the intensities of the Raman and Auger peaks, as well as the asymmetry parameter ξ for the Auger-PCI signals.

Table S1: Values of constrained parameters. All values are given in eV. The starting position $E_{R,0}$ corresponds to a photon energy of 2474 eV.

	**Plasmon**		**Lifetime Broadening**	**Auger**	**Raman**	**Multiplet**
**QD**	E_c	FWHM	Γ_L	E_0 ($^1D_2$)	$E_{R,0}$ ($^1D_2$)	Δ($^1D_2$ - $^1S_0$)
**DL1**	2097.53	24.41	1.0	2118.6	2116.8	8.0
**DL3**	2098.54	16.28	1.0	2117.9	2117.3	8.0
**DL7**	2098.65	17.49	1.0	2117.8	2116.3	8.0
**DL11**	2099.07	17.32	1.0	2118.25	2115.86	7.95
**DL15**	2098.27	17.90	0.92	2117.7	2116.8	8.0

According to the core-hole clock approach, charge transfer times can then be calculated according to the following equation based on the intensity ratio between the Raman and Auger channels:

$$\tau_{CT} = \tau_{CH} f_R \tag{S1}$$

using the Raman fraction f_R:

$$f_R = \frac{I_{Raman}}{I_{Auger}} \tag{S2}$$

and $\tau_{CH} = 1.26$ fs, which is the core-hole lifetime of the S 1s hole [19].

6 Model and Implementation of Post-Collision Interaction Line Shape

The PCI line shape is implemented according to an approximation by Paripás et. al. [20] using the Eikonal approach. The line shape $y(\epsilon)$ at relative energy ϵ is given by:

$$y(\epsilon) = y_0 \frac{k(\xi, \epsilon)}{1 + \epsilon^2} \tag{S3}$$

with

$$k(\xi, \epsilon) = \frac{\pi\xi}{\sinh(\pi\xi)} \exp(-2\xi \arctan(\epsilon))) \tag{S4}$$

and:

$$\epsilon = \frac{E - E_0}{0.5\Gamma_L} \tag{S5}$$

where $y_0(\epsilon)$ is a constant at a given interaction kinematics, Γ_L is the natural linewidth determined by the core-hole lifetime, E is the kinetic energy of the Auger electron, and E_0 is its unperturbed value (without PCI effects). The magnitude of the PCI effect and, thus, the asymmetry of the line is given by the asymmetry parameter ξ and depends on the kinematics of the interaction between photoelectron and Auger electron:

$$\xi = \frac{1}{v_{12}} - \frac{1}{v_1} \tag{S6}$$

where $v_1 = ||\vec{v_1}||$ and $v_{12} = ||\vec{v_1} - \vec{v_2}||$, where $\vec{v_1}$ and $\vec{v_2}$ are the velocities of the Auger electron and photoelectron, respectively.

To account for instrumental broadening effects, the aforementioned line shape is numerically convoluted with a Gaussian profile using SciPy. Thus, an additional Gaussian broadening parameter is added to the model.

To model the evolution of the PCI parameters ξ for the different nanoparticles as a function of photon

energy, Equation S6 is modified to the situation for below-ionization photoelectrons in solids, by introducing the relative permittivity ϵ_r and effective electron mass m_e^*. The velocities are expressed as a function of the kinetic energy as $v = \sqrt{\frac{2E_{kin}}{m_e^*}}$. While the kinetic energy of the Auger electron is known (E_0), the "kinetic energy" of the photoelectron (note that it is still within the bound continuum and, thus, not strictly a photoelectron) is expressed depending on a threshold energy E_T. Note that the equations of the given PCI framework are expressed in terms of atomic units, so that energies in Equation S6 have to be given in units of Hartree:

$$\xi = \frac{1}{\epsilon_r}\left(\frac{1}{\sqrt{\frac{2}{m_e^*}(h\nu - E_T)} - \sqrt{\frac{2}{m_e^*}E_0}} - \frac{1}{\sqrt{\frac{2}{m_e^*}E_0}}\right) \tag{S7}$$

$$\xi = \frac{1}{\epsilon_r\sqrt{\frac{2}{m_e^*}}}\left(\frac{1}{\sqrt{h\nu - E_T} - \sqrt{E_0}} - \frac{1}{\sqrt{E_0}}\right) \tag{S8}$$

The obtained parameters from the fit are shown in Table S2 for all QDs. As this model is applied to the ξ data and uses E_0, both obtained from the previous fitting of the resonant Auger spectra, the uncertainties given here are severely underestimated, as the uncertainties from the heavily constrained resonant Auger fitting are not reliable and were thus not properly propagated. For calculating ϵ_r, 0.28 was taken for the electron effective mass [21].

Table S2: Obtained parameters from fitting ξ as a function of the photon energy for the differently sized QDs.

QD	E_T	ϵ_r
**DL1**	2471.41 ± 0.03 eV	5.06 ± 0.16
**DL3**	2471.35 ± 0.06 eV	6.51 ± 0.41
**DL7**	2471.63 ± 0.01 eV	8.46 ± 0.39
**DL11**	2471.50 ± 0.03 eV	10.67 ± 0.63
**DL15**	2471.63 ± 0.02 eV	9.26 ± 0.50

References

1. Gorgoi, M.; Svensson, S.; Schäfers, F.; Öhrwall, G.; Mertin, M.; Bressler, P.; Karis, O.; Siegbahn, H.; Sandell, A.; Rensmo, H.; Doherty, W.; Jung, C.; Braun, W.; Eberhardt, W. *Nucl. Instrum. Methods Phys. Res., Sect. A* **2009**, *601*, 48–53. doi:10.1016/j.nima.2008.12.244.

2. Schäfers, F. *J. Large-Scale Res. Facil.* **2016**, *2*, A96–A96. doi:10.17815/jlsrf-2-92.

3. Harris, C. R.; Millman, K. J.; van der Walt, S. J.; Gommers, R.; Virtanen, P.; Cournapeau, D.; Wieser, E.; Taylor, J.; Berg, S.; Smith, N. J.; Kern, R.; Picus, M.; Hoyer, S.; van Kerkwijk, M. H.; Brett, M.; Haldane, A.; del Río, J. F.; Wiebe, M.; Peterson, P.; Gérard-Marchant, P.; Sheppard, K.; Reddy, T.; Weckesser, W.; Abbasi, H.; Gohlke, C.; Oliphant, T. E. *Nature* **2020**, *585*, 357–362. doi:10.1038/s41586-020-2649-2.

4. Virtanen, P.; Gommers, R.; Oliphant, T. E.; Haberland, M.; Reddy, T.; Cournapeau, D.; Burovski, E.; Peterson, P.; Weckesser, W.; Bright, J.; van der Walt, S. J.; Brett, M.; Wilson, J.; Millman, K. J.; Mayorov, N.; Nelson, A. R. J.; Jones, E.; Kern, R.; Larson, E.; Carey, C. J.; Polat, I.; Feng, Y.; Moore, E. W.; VanderPlas, J.; Laxalde, D.; Perktold, J.; Cimrman, R.; Henriksen, I.; Quintero, E. A.; Harris, C. R.; Archibald, A. M.; Ribeiro, A. H.; Pedregosa, F.; van Mulbregt, P. *Nat. Methods* **2020**, *17*, 261–272. doi:10.1038/s41592-019-0686-2.

5. Hunter, J. D. *Comput. Sci. Eng.* **2007**, *9*, 90–95. doi:10.1109/MCSE.2007.55.

6. LMFIT: Non-Linear Least-Square Minimization and Curve-Fitting for Python. https://zenodo.org/records/11813 (accessed 2024-05-10). doi:10.5281/zenodo.11813.

7. Yu, W. W.; Qu, L.; Guo, W.; Peng, X. *Chem. Mater.* **2003**, *15*, 2854–2860. doi:10.1021/cm034081k.

8. Wang, F.; Tang, R.; Buhro, W. E. *Nano Lett.* **2008**, *8*, 3521–3524. doi:10.1021/nl801692g.

9. Naughton, M. S.; Kumar, V.; Bonita, Y.; Deshpande, K.; Kenis, P. J. A. *Nanoscale* **2015**, *7*, 15895–15903. doi:10.1039/C5NR04510J.

10. Murray, C. B.; Norris, D. J.; Bawendi, M. G. *J. Am. Chem. Soc.* **1993**, *115*, 8706–8715. doi:10.1021/ja00072a025.

11. Dake, L. S.; Baer, D. R.; Zachara, J. M. *Surf. Interface Anal.* **1989**, *14*, 71–75. doi:10.1002/sia.740140115.

12. Islam, R.; Rao, D. R. *J. Electron Spectrosc. Relat. Phenom.* **1996**, *81*, 69–77. doi:10.1016/0368-2048(95)02551-0.

13. Sloboda, T.; Johansson, F. O. L.; Kammlander, B.; Berggren, E.; Svanström, S.; Fernández, A. G.; Lindblad, A.; Cappel, U. B. *RSC Adv.* **2022**, *12*, 31671–31679. doi:10.1039/D2RA06091D.

14. Klasson, M.; Berndtsson, A.; Hedman, J.; Nilsson, R.; Nyholm, R.; Nordling, C. *J. Electron Spectrosc. Relat. Phenom.* **1974**, *3*, 427–434. doi:10.1016/0368-2048(74)80029-0.

15. Wei, H.; Zhou, J.; Zhang, L.; Wang, F.; Wang, J.; Jin, C. *J. Nanomater.* **2015**, *2015*, e764712. doi:10.1155/2015/764712.

16. Li, D.; Bancroft, G. M.; Kasrai, M.; Fleet, M. E.; Feng, X. H.; Tan, K. H.; Yang, B. X. *J. Phys. Chem. Solids* **1994**, *55*, 535–543. doi:10.1016/0022-3697(94)90052-3.

17. Talapin, D. V.; Mekis, I.; Götzinger, S.; Kornowski, A.; Benson, O.; Weller, H. *J. Phys. Chem. B* **2004**, *108*, 18826–18831. doi:10.1021/jp046481g.

18. Cho, D.-Y.; Xi, L.; Boothroyd, C.; Kardynal, B.; Lam, Y.-M. *Sci. Rep.* **2016**, *6*, 22818. doi:10.1038/srep22818.

19. Campbell, J. L.; Papp, T. *At. Data Nucl. Data Tables* **2001**, *77*, 1–56. doi:10.1006/adnd.2000.0848.

20. Paripás, B.; Vitéz, G.; Víkor, G.; Tőkési, K.; Caló, A.; Sankari, R.; Huttula, M.; Aksela, S.; Aksela, H. *J. Phys. B: At., Mol. Opt. Phys.* **2004**, *37*, 4507. doi:10.1088/0953-4075/37/22/009.

21. Miklosz, J. C.; Wheeler, R. G. *Phys. Rev.* **1967**, *153*, 913–923. doi:10.1103/PhysRev.153.913.
